# Conversion rates in robotic thyroid surgery: A systematic review and meta‐analysis

**DOI:** 10.1002/rcs.2427

**Published:** 2022-06-05

**Authors:** Barbara Martino, Letizia Nitro, Loredana De Pasquale, Paolo Lozza, Alberto Maccari, Luca Castellani, Matilde Piazzoni, Matteo Cardellicchio, Antonio Mario Bulfamante, Carlotta Pipolo, Giovanni Felisati, Alberto Maria Saibene

**Affiliations:** ^1^ Otolaryngology Unit Santi Paolo e Carlo Hospital Department of Health Sciences Università Degli Studi di Milano Milan Italy; ^2^ Thyroid and Parathyroid Surgery Service, Otolaryngology Unit, Santi Paolo e Carlo Hospital Department of Health Sciences Università Degli Studi di Milano Milan Italy

**Keywords:** adverse events, complications, hemithyroidectomy, minimally invasive surgery, robotic surgery, thyroidectomy

## Abstract

**Objective:**

To define the conversion risk to open procedure during robot‐assisted thyroid surgery (RATS) identifying potential specific subclasses of procedures or accesses at higher conversion risk.

**Methods:**

In a PRISMA‐compliant framework, all original prospective studies providing RATS conversion rates from multiple databases were pooled in a random‐effects meta‐analysis. Conversion rates were compared between different typologies of thyroid surgery and robotic access.

**Results:**

13 studies were deemed eligible. Four conversions from two studies were reported out of 398 procedures. No significant heterogeneity was observed (Cochran's *Q p* = 0.932; *I*2 = 0%). The pooled conversion rate was 1% (95% confidence interval, 0.1%–2%). The ANOVA‐Q test failed to show significant differences when comparing type of thyroid surgery or robotic access (respectively *p* = 0.766 and *p* = 0.457).

**Conclusion:**

While the conversion rate appears consistently low across studies, prospective data collection and systematic reporting of procedural complications are required for framing high‐risk procedures and accesses.

## 1 INTRODUCTION

Minimally invasive surgical techniques, whether robot‐ or endoscope‐assisted, have a known risk of conversion to open irrespectively of the surgical area of application.^1^, ^2^, ^3^

While robotic assistance has been first employed for thyroid surgery later than in other anatomical compartments,^4^ its use soon became widespread. Reports of conversions, therefore, grew in numbers also for thyroid surgery. In robotic‐assisted thyroid surgery (robot‐assisted thyroid surgery (RATS), known conversion had been required, among other causes, for excessive bleeding, previously undetected neoplastic infiltration or unexpected disease extension, or technical issues.^5^, ^6^, ^7^

Despite the potential need for conversion being recognized in most case series, conversion rates are reported inconsistently in the literature and span from large case series with no conversion^8^ to significantly preliminary smaller series with rates higher than 15%.^9^

To the authors' knowledge, no study has systematically explored the risk of conversion in RATS or addressed whether different RATS procedures (e.g. Total thyroidectomy ,TT, hemithyroidectomy (HT), or radicalisation thyroidectomy) or approaches (e.g. transoral, facelift, gasless transaxillary, retroauricolar, robotic‐assisted breast‐axillo insufflation thyroidectomy) hold significantly different conversion rates. Defining such risk of conversion appears pivotal, as the need for a neck incision for controlling the surgical field, despite not hindering the procedure outcomes, nullifies the major advantage of RATS, that is, the scarless or near‐scarless approach.^10^

This systematic review and meta‐analysis aims at delineating the risk of conversion in RATS and assessing whether specific subclasses of procedures or accesses should be regarded at higher risk.

## 2 METHODS

This review was registered in the International Prospective Register of Systematic Reviews under the number CRD42021277928.

### 2.1 Search strategy

A systematic review and meta‐analysis was conducted between 12 September 2021, and 20 January 2022, according to the Preferred Reporting Items for Systematic Reviews and Meta‐analyses reporting guidelines.^11^ We completed systematic electronic searches for studies written in English, Italian, German, French, or Spanish published until the search date that reported original data obtained from humans and focussed entirely or partly on RATS in humans.

On 23 September 2021, we searched MEDLINE, Embase, Web of Science, Scopus, Cochrane Library, and http://ClinicalTrials.gov databases using wide search strategies for thyroid‐, thyroid surgery‐, and robot‐related terms. The detailed search strategy with the number of unique items retrieved from each database is available in Table 1.

**TABLE 1:** Databases, keys, and number of unique results for the initial search

Database	Key	Results
Cochrane library	(Thyroid OR thyroidectomy OR hemithyroidectomy) AND (robot OR robotic OR robot‐assisted OR ‘robot assisted’) in all text ‐ (word variations have been searched)	69
Medline	(Thyroid OR thyroidectomy OR hemithyroidectomy) AND (robot OR robotic OR robot‐assisted OR ‘robot assisted’)	720
Clinicaltrials.gov	(Thyroid OR thyroidectomy OR hemithyroidectomy) AND (robot OR robotic OR robot‐assisted OR ‘robot assisted’)	20
Scopus	TITLE‐ABS‐KEY ((thyroid OR thyroidectomy OR hemithyroidectomy) AND (robot OR robotic OR ‘robot AND assisted’))	540
Embase	(thyroid:ti,ab, kw OR thyroidectomy:ti,ab, kw OR hemithyroidectomy:ti,ab,kw) AND (robot:ti,ab, kw OR robotic:ti,ab, kw OR ‘robot assisted’:ti,ab,kw)	826
Web of science	(Thyroid OR thyroidectomy OR hemithyroidectomy) AND (robot OR robotic OR robot‐assisted OR ‘robot assisted’) (topic)	782

We included any study dealing with RATS in humans. We excluded cadaver studies, meta‐analyses, systematic and narrative reviews, and case reports, though references from review articles were hand‐checked for additional potentially relevant studies. No minimum study population was required. We included only prospective studies that explicitly reported conversion rates (even if nil) and specified the robotic technique of choice and the type of thyroid surgeries that had been performed.

Abstracts and full texts were reviewed in duplicate by different authors (B.M. and L.N.). To maximise the rate of inclusivity in the early stages of the review, at the abstract stage, we included all studies deemed eligible by at least one rater. Then, during the full‐text review stage, disagreements were resolved by consensus between raters.

### 2.2 Patient/population, intervention, comparison, outcomes, timing, studies (PICOTS) criteria

The Patient/population, intervention, comparison, outcomes, timing, studies (PICOTS) criteria for the present review were as follows:

P: patients with thyroid disease candidate to RATS
I: RATS
C: comparison between different typologies of available robot‐assisted approaches and thyroid surgeries (total thyroidectomy, subtotal thyroidectomy, hemithyroidectomy, completion thyroidectomy)
O: RATS conversion rate into open surgery
T: intraoperative events only
S: all prospective original studies except case reports

3

For each included article, we recorded study type, country of origin, number of RATS cases, overall number of patients included in the study, RATS patients' female to male ratio and age, thyroid and nodule size, and body mass index ,BMI criteria for eligibility to RATS, final histology, number of RATS procedure according to typology of thyroid surgery, number and type of neck dissections during RATS, number of procedures according to RATS access type, number of conversion and details on converted procedures, and conversion rate with other closed techniques (where available).

Selected studies were assessed for both quality and methodological bias according to the National Heart, Lung, and Blood Institute Study Quality Assessment Tools (NHI‐SQAT).^12^ Articles were rated in duplicate by two authors (B.M. and L.N), with disagreements resolved by consensus. Items were rated as good if they fulfiled at least 80% of the items required by the NHI‐SQAT, fair if they fulfiled between 50% and 80% of the items, and poor if they fulfiled less than 50% of the items, respectively.

Also, the level of evidence was scored according to the Oxford Centre for Evidence‐based Medicine (OCEBM) level of evidence guide.^13^ For clinical trials, bias was assessed with the revised Cochrane risk of bias tool for randomized trials.^14^

Articles rated as being of fair or good quality according to the NHI‐SQAT were selected for meta‐analysis. The pooled frequency of conversion to open surgery with 95% confidence intervals was assessed using a Der Simonian ‐ Laird random‐effects model. Conversion rates were also compared according to the robotic access used and the type of thyroid surgery being performed via the ANOVA‐Q test, again in a random‐effects model. The between‐study heterogeneity was assessed by Cochran's *Q* and I2 statistics. Publication bias was assessed graphically via the funnel plot method and Egger's and Begg's test.

All search results, abstract and article selection, data extraction, and descriptive statistics were performed with the Google Sheets web application (Google LLC, Mountain View, CA, USA). The meta‐analysis was performed using the freeware software Openmeta [Analyst] (built 12/3/2013; Brown University, Providence, RI, USA) and Prometa (version 3.0; IDoStatistics, Italy).

## 4 RESULTS

### 4.1 Search results

**FIGURE 1:**
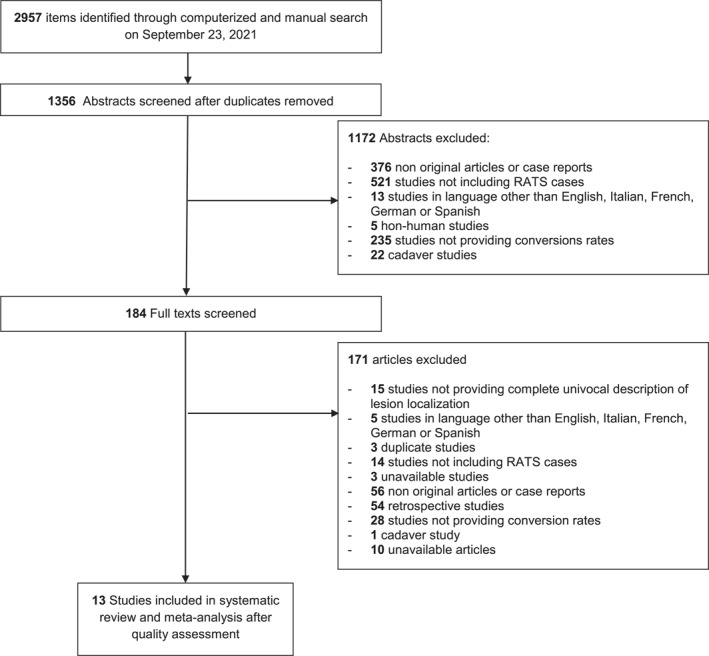
PRISMA‐style flow chart of selection of studies throughout the systematic review and meta‐analysis

Among the 1356 unique research items initially identified, a total of 184 articles were selected to undergo full‐text evaluation. Ultimately, 13 studies published between 2010 and 2019 were retained for further analysis (see Figure 1).

Table 2 reports the characteristics and demographics of the included studies. 11 articles were prospective cohort studies,^15^, ^16^, ^17^, ^18^, ^19^, ^20^, ^21^, ^22^, ^23^, ^24^, ^25^ one was a prospective case‐control study^26^ and one was a randomized controlled trial, RCT.^27^ Four studies were performed in Europe (Great Britain, Greece, Italy, and Romania), four in the United States of America, and five in Asia (China, *n* = 2, and South Korea, *n* = 3). All studies were rated as level 2 studies according to the OCEBM scale. According to the NHI‐SQAT, seven articles were rated as good‐quality studies, six articles were rated as fair‐quality studies, and no articles were rated as poor‐quality studies. Most articles lacked ample information to support the comparability of patients. No other significant biases were identified. For the single randomized trial, analysis via the Cochrane tool suggested a high risk of bias in the randomisation process and some concerns in deviation from intended interventions and low risk of bias in all other domains, with some concerns in the overall risk of bias of the study.

**TABLE 2:** Characteristics and demographics of the included Studies

Study	Article‐type	Country of origin	OCEBM rating	NHI‐SQAT rating	No. Of procedures		Female to male ratio for RATS group	Age for RATS group (years)	Eligibility criteria for RATS	
					RATS procedures	Total procedures			Nodule/gland characteristics	BMI (kg/m2)
Arora et al, 2016^26^	PCCS	GB	2	G	16	32	15:1	42 ± 10.8	Solitary nodule< 6 cm	n/a
Axente et al, 2013^15^	PCS	Romania	2	F	50	50	49:1	47.5 ± 15.24	Uni‐ or bilateral nodules <6 cm and no suspicion of malignancy	n/a
Chai et al, 2016^16^	PCS	South Korea	2	F	27	54	27:1	36.0 ± 8.3	n/a	n/a
Duke et al, 2016^17^	PCS	USA	2	G	90	102	89:1	41.9 ± 13.1	Largest nodule ≤4 cm.	<30
He et al, 2016^27^	RCT	China	2	F	50	100	41:9	40.9 ± 9.8	Intrathyroidal papillary carcinoma <10 mm, lobe volume <40 ml	<30
Kandil et al, 2011>^18^	PCS	USA	2	F	5	5	3:2	36.2 ± 5.56	n/a	n/a
Kandil et al, ^2014^ ^19^	PCS	USA	2	G	12	12	12:0	45 ± 4.43	Nodule <4 cm	<30
Kim et al, 2015^20^	PCS	South Korea	2	G	10	10	10:0	35.1 ± 9.6	n/a	n/a
Kiriakopoulos and linos, 2012^21^	PCS	Greece	2	F	8	12	6:2	38.8 ± 8.9	Nodules <5 cm	<30
Lang and chow, 2010^22^	PCS	China	2	G	7	46	7:0	Median 43.4, range 20.2–54.7	Dominant nodule <4 cm in benign cases and <2 cm in potentially malignant cases	n/a
Lee et al, 2015^23^	PCS	South Korea	2	G	76	280	62:14	43.6 ± 11.8	n/a	n/a
Prete et al, 2019^24^	PCS	Italy	2	G	12	12	12:0	Mean 44.9, range 31–63	Nodules <5 cm (<1 cm if suspect for differentiated ca), lobe <7 cm	<30
Rodriguez et al, 2011^25^	PCS	USA	2	F	35	35	30:5	Mean 42.15, range 3–79, median 37	n/a	n/a

*Note*: Age is reported as mean ± standard deviation unless otherwise stated.

Abbreviations: BMI, body mass index.; F, fair; G, good; lung, and blood institute study quality assessment tools; NHI‐SQAT, national heart; OCEBM, Oxford centre for evidence‐based medicine; PCCS, prospective case‐control study; PCS, prospective cohort study; RATS, robot‐assisted thyroid surgery; RCT, randomized controlled trial; SD, standard deviation.

The 13 included studies reported 398 RATS procedures on individual patients out of a total of 750 procedures. There was a clear female prevalence across studies (363 female patients and 35 male patients were included in the studies), with female sex being an explicit inclusion criterion in 2 studies. RATS patients were on average in their third or fourth decade in all studies. Nine studies provided variable nodular or glandular dimensional criteria for eligibility to RATS, while a BMI lower than 30 kg/m^2^ was an eligibility criterion for RATS in 5 studies.

Two hundred 45 RATS procedures were total thyroidectomies, 138 were hemithyroidectomies and 15 were subtotal thyroidectomies. 89 patients underwent concomitant robot‐assisted central compartment neck dissection (ND), while no lateral compartment dissection was performed. In 87 patients a bilateral axillo‐breast access (BABA) was used, in 102 patients a retroauricular facelift approach (RFA) was used and in 209 a transaxillary gasless approach (TGA) was used. Each study employed a specific RATS approach and no intra‐study comparison for RATS approaches was available. Two studies^15^ ^,^ ^25^ reported a total of 4 conversions into open procedures, all occurring during TGA total thyroidectomies. One conversion was due to excessive bleeding in a female patient, another one was due to unexpected high glandular volume (multinodular goitre) and two were due to previously undetected significant tumour extension (specifically, a papillary cancer invading the cricothyroid area and a follicular carcinoma with cranial extension). All converted procedures were completed via a midline neck incision and without any further complication. Data on procedures, approaches, and conversion rates are reported in Table 3.

**TABLE 3:** Data on procedures, approaches, and conversion rates of the included studies

Study	RATS procedure by type (n)			Associated robot‐assisted ND		RATS procedures by access			Conversion rate
	TT	HT	ST	CND	LND	BABA	RFA	TGA	
Arora et al, 2016^26^	16	0	0	0	0	0	0	16	0:16
Axente et al, 2013^15^	9	33	8	0	0	0	0	50	1:50
Chai et al, 2016^16^	27	0	0	27	0	27	0	0	0:27
Duke et al, 2016^17^	12	78	0	1	0	0	90	0	0:90
He et al, 2016^27^	50	0	0	50	0	50	0	0	0:50
Kandil et al, 2011^18^	0	0	5	0	0	0	0	5	0:5
Kandil et al, 2014^19^	2	10	0	0	0	0	12	0	0:12
Kim et al, 2015^20^	9	1	0	9	0	10	0	0	0:10
Kiriakopoulos and linos, 2012^21^	3	3	2	1	0	0	0	8	0:8
Lang and chow, 2010^22^	4	3	0	1	0	0	0	7	0:7
Lee et al, 2015^23^	76	0	0	0	0	0	0	76	0:76
Prete et al, 2019^24^	2	10	0	0	0	0	0	12	0:12
Rodriguez et al, 2011^25^	35	0	0	0	0	0	0	35	3:35

Abbreviations: BABA, bilateral axillo‐breast approach; CND, central ND; HT, hemithyroidectomy; LND, lateral ND; ND, neck dissection; RATS, robot‐assisted thyroid surgery; RFA, retroauricolar facelift approach; ST, subtotal thyroidectomy; TGA, transaxillary gasless approach; TT, total thyroidectomy.

RATS was compared with open approaches in 4 studies and with endoscopic thyroidectomy in two studies. A single conversion from endoscopic to open was reported over 43 overall endoscopic procedures.

Final histology reports for included patients are reported in Table 4.

**TABLE 4:** Final histological diagnoses in the reviewed articles

Study	Final histologic diagnoses	
	Benign	Malignant
Arora et al, 2016^26^	Multinodular goitre (*n* = 3), follicular adenoma within multinodular goitre (*n* = 2), thyroid cyst (*n* = 1), dominant nodule in goitre (*n* = 1), colloid nodule (*n* = 2)	Papillary cancer (*n* = 2), papillary microcarcinoma (*n* = 1), papillary carcinoma within goitre (*n* = 1)
Axente et al, 2013^15^	Multinodular goitre (*n* = 25), follicular adenoma (*n* = 13), toxic adenoma (*n* = 2), hurtle cell adenoma (*n* = 2), graves' disease (*n* = 2), papillary adenoma (*n* = 2), nodular autoimmune thyroiditis (*n* = 3), diffuse goitre (*n* = 1)	Papillary cancer (*n* = 2)
Chai et al, 2016^16^	None	Malignant (papillary) *n* = 27
Duke et al, 2016^17^	Adenoma (*n* = 29), multi‐nodular goitre (*n* = 39), toxic adenoma (*n* = 1), thyroid cyst (*n* = 1), no pathologic findings (*n* = 3)	Papillary carcinoma (*n* = 21) papillary microcarcinoma (*n* = 5), follicular carcinoma (*n* = 4), sclerosing mucoepidermoid carcinoma (*n* = 1).
He et al, 2016^27^	None	Papillary microcarcinoma (*n* = 50)
Kandil et al, 2011^18^	Graves' disease (*n* = 5)	None
Kandil et al, 2014^19^	Hyperplasia (*n* = 7), hashimoto thyroiditis (*n* = 2)	Follicular carcinoma (*n* = 2), papillary carcinoma (*n* = 1)
Kim et al, 2015^20^	Nodular hyperplasia (*n* = 1)	Papillary carcinoma (*n* = 9)
Kiriakopoulos and linos, 2012^21^	Toxic adenoma (*n* = 3), multinodular goitre (*n* = 2)	Papillary carcinoma (*n* = 3)
Lang and chow, 2010^22^	Nodular hyperplasia (*n* = 6)	Papillary carcinoma (*n* = 1)
Lee et al, 2015^23^	None	Unspecified malignant histology (*n* = 76)
Prete et al, 2019^24^	Unspecified benign histology (*n* = 7)	Papillary microcarcinoma (*n* = 5)
Rodriguez et al, 2011^25^	Unspecified benign histology (*n* = 12)	Follicular carcinoma (*n* = 19), hurtle cell carcinoma (*n* = 1), papillary carcinoma (*n* = 3)

All articles of fair or good quality according to the NHI‐SQAT were included as no significant methodological bias emerged, therefore all 13 articles were included in the final meta‐analysis.

**FIGURE 2:**
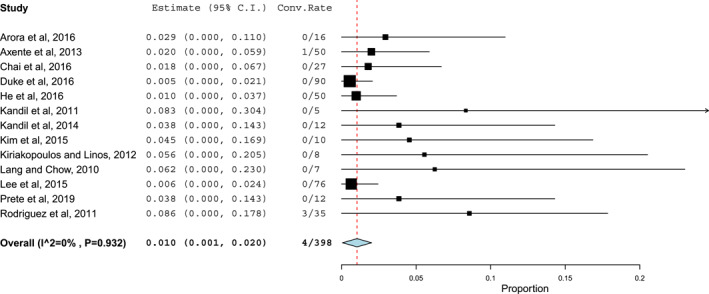
The pooled conversion rate in patients undergoing robot‐assisted thyroid surgery (RATS). Effects and summaries were calculated using a random‐effect model weighted by the study population

The funnel plot method and Begg's test suggested the presence of some degree of publication bias in the published literature (*p* = 0.458), while no significant heterogeneity was observed between studies (Cochran's *Q p* = 0.932; *I*2 = 0%). The pooled conversion rate for patients undergoing RATS was 1% (95% confidence interval, 0.1%–2%) (see Figure 2), with an effect size of 0.06 (95% confidence interval, 0.03–0.1). As the ANOVA‐Q test failed to show significant differences when comparing patients for the type of thyroid surgery performed or robotic access used (respectively *p* = 0.766 and *p* = 0.457), no subgroup analyses were performed.

Due to the low numbers of endoscopic procedures provided as a comparison in the reviewed articles, a meta‐analytic comparison between different minimally invasive thyroidectomies choices was deemed too biased to provide adequate supporting clinical evidence. Analogously, the heterogeneity and reporting inconsistency in patient selection criteria prevented a targeted meta‐analytic subgroup comparison.

## 5 DISCUSSION

To the authors' knowledge, this is the first systematic review to address specifically the risk of RATS conversion into open‐neck procedures. Despite the small effect size, we found that the conversion rate is consistent between different studies and with the 1% pooled rate emerging from our meta‐analysis. This issue, intrinsic to all minimally invasive procedures irrespective of the surgical site, has not been addressed by already published review works, either because they were focussed on other safety features^28^ or because they simply explored the differences between robotic and open procedures, the latter being unaffected per se by conversions.^29^ ^,^ ^30^ Analogously, the same risk has not been specifically assessed also for endoscopic thyroidectomies procedures, with review works and meta‐analytic comparison focussing again on different aspects of the surgical procedures and other patient outcomes.^31^ ^,^ ^32^

The 1% conversion rate stemmed from four different events during TGA total thyroidectomies reported in two studies from different groups.^15^ ^,^ ^25^ These data were recovered from a set of 398 robot‐assisted procedures in 13 average‐to‐good methodological quality small‐scale prospective studies. Despite the good evidence level and methodological consistency, the relatively small scale of studies determined a moderate degree of publication bias, which should be hopefully covered by future larger‐scale studies. The procedures taken into account cover most types of thyroid surgery types (ST, HT, and TT, with a clear predominance of the last, accounting for 62% of procedures), with or without ND, performed with three distinct access types (BABA, RFA, and TGA, the last accounting alone for 53% of procedures). If we examine the four conversion events, it's interesting to observe that a more thorough preoperative planning potentially could avoid the three events due to unexpectedly relevant tumour extension or goitre volume, while the bleeding event remains unforeseeable. Though these data are still scarce, careful preoperative imaging, especially in case of malignancy or high volume goitres, might be beneficial on the conversion rates.

Given the huge impact of the scarless or near‐scarless approach of RATS on patients' surgical preferences, a clearer definition of conversion risks is of the utmost importance for informed consent purposes. This systematic review allows for a more data‐driven patient consent going beyond single‐study results, which show a significant variation in conversion rates, especially in retrospective case series. Even if we take into account that several high‐volume centres have considerable experience in RATS, it still comes as surprising to see conversions raising from 0% in three case series with *n* > 500^8^ ^,^ ^33^ ^,^ ^34^ to 16.6–33.3% in small, either preliminary or non‐thyroid‐specific, case series with *n* < 10.^9^ ^,^ ^35^

Even if we take into account the experience of high‐volume tertiary centres or the pilot experience on specific accesses or high‐risk patient classes, the mere existence of such a considerable gap in reported conversion rates claims the possibility of a reporting bias. On the basis of this potential bias, this meta‐analysis was based only on prospective studies, thus allowing for a higher level of evidence and also for avoiding duplicated results that may be generated by partially overlapping case series presented in different articles or multi‐centre studies.

On the other hand, the small number of prospective studies available in the literature and their relatively small sample sizes prevented us from drawing conclusions on the potential differential risk between different types of RATS procedures or accesses. Although TT might indeed hold a higher conversion risk than HT, being thyroidectomy and TGA respectively the most common procedure and access in this meta‐analysis, it's not surprising that all conversion cases belong to these groups. Analogously, the sample size is too small to draw any reliable comparison with the conversion rate for endoscopic procedures and the allocation bias to RATS versus endoscopy‐assisted procedures or open procedures might be considerable, as the single RCT included demonstrates. Furthermore, the relatively small size of included studies determines a more considerable publication bias. Nevertheless, these biases were considered too low to hinder the overall value of our conclusions. Another limitation of this meta‐analysis stems from the heterogeneity of RATS eligibility criteria in terms of nodule/thyroid volume and the inconstant reporting of BMI criteria, which do not allow to draw any conclusion on which thyroid‐ and patient‐specific characteristics determine a higher risk of conversion. Last, databases searches didn't locate any eligible stud reporting conversion rates for transoral robotic thyroidectomy (TORT), so this approach was not covered in our analysis or included in any subgroup evaluation. Nevertheless, a recent wide retrospective multicentric review suggested TORT has similar conversion rates as those emerging from our systematic review, around 0.7%.^36^

## 6 CONCLUSION

By providing a novel insight on the conversion risk in RATS, this meta‐analysis calls nevertheless for greater attention to this often neglected surgical adverse event. Only routine implementation of a common and unbiased reporting system into prospective multicentric studies might allow for better defining patient groups, RATS procedures, and accesses at higher conversion risk, and providing a sounder risk assessment. Irrespective of future studies, the conversion risk should be adequately discussed with patients in everyday practice, given its impact on the secondary outcomes of RATS. Even if aggregate conversion rates are low, an average 1% risk of neck scar is worth exploring with patients who mostly see RATS as the scarless answer to their thyroid disease.

## CONFLICT OF INTEREST

The authors have no potential conflict of interest or financial disclosures pertaining to this article.

## Data Availability

All data pertaining to this meta‐analysis are available from the authors upon reasonable request.
